# Tensor-Decomposition-Based Unsupervised Feature Extraction in Single-Cell Multiomics Data Analysis

**DOI:** 10.3390/genes12091442

**Published:** 2021-09-18

**Authors:** Y-h. Taguchi, Turki Turki

**Affiliations:** 1 Department of Physics, Chuo University, Tokyo 112-8551, Japan; 2 Department of Computer Science, King Abdulaziz University, Jeddah 21589, Saudi Arabia; tturki@kau.edu.sa

**Keywords:** tensor decomposition, feature extraction, single-cell, multiomics data

## Abstract

Analysis of single-cell multiomics datasets is a novel topic and is considerably challenging because such datasets contain a large number of features with numerous missing values. In this study, we implemented a recently proposed tensor-decomposition (TD)-based unsupervised feature extraction (FE) technique to address this difficult problem. The technique can successfully integrate single-cell multiomics data composed of gene expression, DNA methylation, and accessibility. Although the last two have large dimensions, as many as ten million, containing only a few percentage of nonzero values, TD-based unsupervised FE can integrate three omics datasets without filling in missing values. Together with UMAP, which is used frequently when embedding single-cell measurements into two-dimensional space, TD-based unsupervised FE can produce two-dimensional embedding coincident with classification when integrating single-cell omics datasets. Genes selected based on TD-based unsupervised FE are also significantly related to reasonable biological roles.

## 1. Introduction

Single-cell multiomics data analysis is challenging [1]. There are multiple reasons for this issue. First, it inevitably includes too many missing values. In the usual high-throughput sequencing (HTS), the so-called depth can compensate for this problem. Nevertheless, because of the very limited amount of RNA retrieved from individual cells available, “depth” cannot resolve this missing value problem. Second, too many missing values result in apparent diversity. The primary purpose of single-cell analysis is to identify the diversity of individual cells that cannot be recognized by the tissue-level HTS. Although missing values are random, apparently very variant profiles appear from a single profile, which can be recognized if there is a large enough number of reads available. This compels researchers to distinguish between true biological diversity and apparent diversity caused by missing values [2].

Finally, single-cell analysis is computationally challenging. Because there are not many samples in the standard HTS, even if the number of features is large, the overall required computational resources decided by the product between the number of features and the number of samples are very limited. Nonetheless, since the number of samples that is the same as that of cells can be huge in single-cell analysis, single-cell analysis can be computationally very challenging.

To resolve these difficulties, we employed tensor-decomposition (TD)-based unsupervised feature extraction (FE) [3]. Prior to applying TD to multiomics datasets, singular-value decomposition (SVD) was applied to individual omics profiles such that individual omics profiles have common *L* singular-value vectors. Then, *K* omics profiles are formatted as an L×M×K-dimensional tensor, where *M* is the number of single cells. Then, higher-order singular-value decomposition (HOSVD), which is a type of TD, is applied to the tensor. UMAP applied to singular-value vectors attributed to single cells by HOSVD successfully generated two-dimensional embedding, coincident with the known classification of single cells.

## 2. Materials and Methods

### 2.1. Gene Expression Profiles

Two single-cell multiomics datasets were downloaded from GEO using the following two GEO IDs.

#### 2.1.1. GSE154762: Dataset 1

The multiomics dataset [4] retrieved from GEO ID GSE154762, which is denoted as Dataset 1 in this study, is composed of 899 single cells for which gene expression, DNA methylation, and DNA accessibility were measured. These single cells represent human oocyte maturation (Table 1). For gene expression, the file “GSE154762_hO_scChaRM_count _matix.txt.gz” was downloaded from the supplementary file of GEO and was loaded into R [5] using the read.table function in R. For DNA methylation and DNA accessibility, 899 files with the extensions “WCG.bw” and “GCH.bw” were downloaded from the supplementary files of GEO and were loaded into R using the import function in the rtracklayer [6] package in R.

#### 2.1.2. GSE121708: Dataset 2

The multiomics dataset [7] retrieved from GEO ID GSE154762, which is denoted as Dataset 2 in this study, is composed of 852 single cells for which DNA methylation and DNA accessibility were measured, as well as 758 single cells for which gene expression was measured. These single cells represent the four time points of the mouse embryo (Table 2). For gene expression, the file “GSE121650_rna_counts.tsv.gz” was downloaded from the supplementary file of GEO and was loaded into R using the read.table function in R. For DNA methylation and DNA accessibility, 852 files with the extensions “met.tsv.gz” and “acc.tsv.gz” were downloaded from the supplementary file of GEO and were loaded into R using the read.table function in R.

### 2.2. Preprocessing of DNA Methylation Profiles

First, we collected genomic positions for which at least one measurement was performed for at least one single cell (i.e., union). Then, for each genomic position, three integers, −1, 0, and 1, were assigned. When the genomic position was measured in a single cell and its state was methylated (nonmethylated), we attributed 1 (−1) to the genomic position of the single cell. Otherwise (i.e., missing observation), we attributed 0 to the genomic transition in a single cell. $x_{ij2}\in R^{N_{2}\times M}$ was stored as a sparse matrix object using the Matrix [8] package in R because of the large $N_{2}$.

### 2.3. Preprocessing of DNA Accessibility

First, we divided the whole genome into 200 nucleotide regions, and DNA accessibility was summed up within individual regions. These values, which show the summation of DNA accessibility within individual regions, are regarded as DNA accessibility at the individual 200 nucleotide regions, each of which is supposed to approximately correspond to a single nucleosome that is composed of 140-length DNA that wraps around histones and 80-length linker DNA. In this study, these 200 nucleotide regions are called “nucleosome regions”. $x_{ij3}\in R^{N_{3}\times M}$ was stored as a sparse matrix object using the Matrix package in R because of the large $N_{3}$.

### 2.4. TD-Based Unsupervised FE

#### 2.4.1. Reduction of Feature Dimensions

Here, feature denotes gene expression, DNA methylation, or DNA accessibility. Because the features of these three datasets differ from one another, we first applied SVD to these features. Suppose $x_{ijk}\in R^{N_{k}\times M\times K}$ represents the value of the *i*th feature (expression of the *i*th gene, methylation of the *i*th genomic location, or DNA accessibility of the *i*th nucleosome region) at the *k*th single cell of the *k*th omics data (1≤k≤K=3, k=1: gene expression, k=2: DNA methylation, and k=3: DNA accessibility). Applying SVD to $x_{ijk}$, we obtain:

(1) $$x_{ijk}=\sum_{\ell =1}^{L}\lambda_{\ell }u_{\ell ik}v_{\ell jk}$$

where $\lambda_{\ell }$ is the *ℓ*th singular value and $u_{\ell_{i}k}$ and $v_{\ell jk}$ are the *i*th and *j*th components of the *ℓ*th left and right singular-value vectors, respectively. Then, $x_{ijk}$ is transformed to $x_{\ell jk}\in R^{L\times M\times K}$ to have the same (common) feature dimension, *L*, independent of *k*, as:

(2) $$x_{\ell jk}=\sum_{i=1}^{N_{k}}u_{\ell ik}x_{ijk}$$

#### 2.4.2. Data Normalization

Prior to applying SVD to the individual omics profiles in these two datasets, $x_{ijk},\left(k=2,3\right)$, that is DNA methylation and accessibility, of Dataset 1 was normalized such that:

(3) $$\sum_{i=1}^{N_{k}}\left|x_{ijk}\right|=N_{k}$$

whereas $x_{ij1}$, i.e., gene expression, was normalized such that:

(4) $$\begin{array}{ccc} \sum_{i=1}^{N_{K}}x_{ij1} & = & 0 \end{array}$$

(5) $$\begin{array}{ccc} \sum_{i=1}^{N_{k}}x_{ij1}^{2} & = & N_{k} \end{array}$$

for Datasets 1 and 2. The reason why DNA methylation and the accessibility of Dataset 2 were not normalized is because $\sum_{i}\left|x_{ijk}\right|,\left(k=2,3\right)$ is very small in some single cells in Dataset 2. Thus, applying normalization adds significant weight to these single cells with fewer observations and drastically skewed outcomes. To avoid this problem, $x_{ijk},\left(k=2,3\right)$ of Dataset 2 was not normalized.

#### 2.4.3. TD Applied to Dimension-Reduced Multiomics Datasets

HOSVD [3] was applied to the tensor, $x_{\ell jk}$, and we obtained:

(6) $$x_{\ell jk}=\sum_{\ell_{1}=1}^{L}\sum_{\ell_{2}=1}^{M}\sum_{\ell_{3}=1}^{K}G\left(\ell_{1}\ell_{2}\ell_{3}\right)u_{\ell_{1}\ell }u_{\ell_{2}j}u_{\ell_{3}k}$$

where $G\in R^{L\times M\times K}$ is the core tensor that represents the contribution of $u_{\ell_{1}\ell }u_{\ell_{2}j}u_{\ell_{3}k}$ to $x_{\ell jk}$. $u_{\ell_{1}\ell }\in R^{L\times L},u_{\ell_{2}j}\in R^{M\times M},u_{\ell_{3}k}\in R^{K\times K}$ are singular-value matrices and are orthogonal matrices.

### 2.5. Categorical Regression

For categorical regression to test the coincidence between classification shown in Table 1 or Table 2 and singular-value vectors attributed to the *j*th single cells, we performed categorical regression:

(7) $$\begin{array}{ccc} v_{\ell jk} & = & a_{\ell ks}\delta_{js}+b_{\ell k} \end{array}$$

(8) $$\begin{array}{ccc} u_{\ell_{2}j} & = & a_{\ell_{2}s}\delta_{js}+b_{\ell_{2}} \end{array}$$

where *s* denotes one of the classifications shown in Table 1 or Table 2, $a_{\ell ks},b_{\ell k},a_{\ell_{2}s},b_{\ell_{2}}$ are regression coefficients, and $\delta_{js}$ takes the value of 1 when the *j*th single cell belongs to the *s*th classification and 0 otherwise. Categorical regression was performed using the ls function in R. The obtained *p*-values were corrected using the Benjamini-Hochberg (BH) criterion [3]. *ℓ*s or $\ell_{2}$s associated with adjusted *p*-values less than 0.01 were regarded to be coincident with classification.

### 2.6. UMAP

Two-dimensional embedding was performed by UMAP [9]. The umap function implemented in R was used.

### 2.7. Gene Selection

After identifying which $u_{\ell_{2}j}$ coincided with the classification, we needed to identify which $u_{\ell_{1}\ell }$ was associated with the selected $u_{\ell_{2}j}$ by investigating $\left|G(\ell_{1}\ell_{2}\ell_{3})\right|$; $\ell_{1}$s with a larger |G| with the selected $\ell_{2}$ were regarded to be coincident with the classification. Then, the selected $u_{\ell_{1}\ell }$ was converted back to $u_{\ell_{1}i_{1}}$ attributed to genes as:

(9) $$u_{\ell_{1}i}=\sum_{\ell =1}^{L}u_{\ell_{1}\ell }u_{\ell i1}$$

*p*-values can be attributed to genes, *i*, assuming $u_{\ell_{1}i}$ obeys a multiple Gaussian distribution (null hypothesis) as:

(10) $$P_{i}=P_{\chi^{2}}\left[>\sum_{\ell_{1}}{\left(\frac{u_{\ell_{1}i}}{\sigma_{\ell_{1}}}\right)}^{2}\right]$$

where the summation is taken over only the selected $\ell_{1}$s, $P_{\chi^{2}}\left[>x\right]$ is the cumulative $\chi^{2}$ distribution, where the argument is larger than *x*, and $\sigma_{\ell_{1}}$ is the standard deviation. $P_{i}$s were corrected by the BH criterion [3], and *i*s associated with adjusted $P_{i}$ less than 0.01 were selected.

## 3. Results

### 3.1. Dataset 1

We obtained $x_{ij1}\in R^{26500\times 899}$, $x_{ij2}\in R^{26438807\times 899}$, and $x_{ij3}\in R^{15478375\times 899}$. SVD was applied to $x_{ijk}$ with L=10, as in Equation (1). For $x_{ijk},k=2,3$, SVD was performed using the irlba function in the irlba package [10] in R because of the large $N_{k},k=2,3$, as many as ten million. Then, HOSVD was applied to $x_{\ell jk}$, as in Equation (2).

One possible validation to check whether the above procedure works properly is to check whether $v_{\ell jk}$ and $u_{\ell_{2}j}$ are coincident with the classification shown in Table 1. Because the above procedure is fully unsupervised, it is unlikely that $v_{\ell jk}$ and $u_{\ell_{2}j}$ are accidentally coincident with the classification. To quantitatively validate the coincidence between the classification and $v_{\ell jk}$ or $u_{\ell_{2}j}$, we applied categorical regression (see Section 2.5).

Table 3 shows the number of singular-value vectors coincident with the classification shown in Table 1. When SVD was applied to individual omics data, because L=10, the number of singular-value vectors was 10 as well. When *K* omics datasets were integrated and HOSVD was applied, the number of singular-value vectors was KL=10K. Thus, when DNA methylation and accessibility were integrated, 20 singular-value vectors were available. When all three omics data were integrated, 30 singular-value vectors were available. It is obvious that for all five cases, at least one singular-value vector was coincident with the classification. Thus, our strategy was essentially successful.

**Figure 1:**
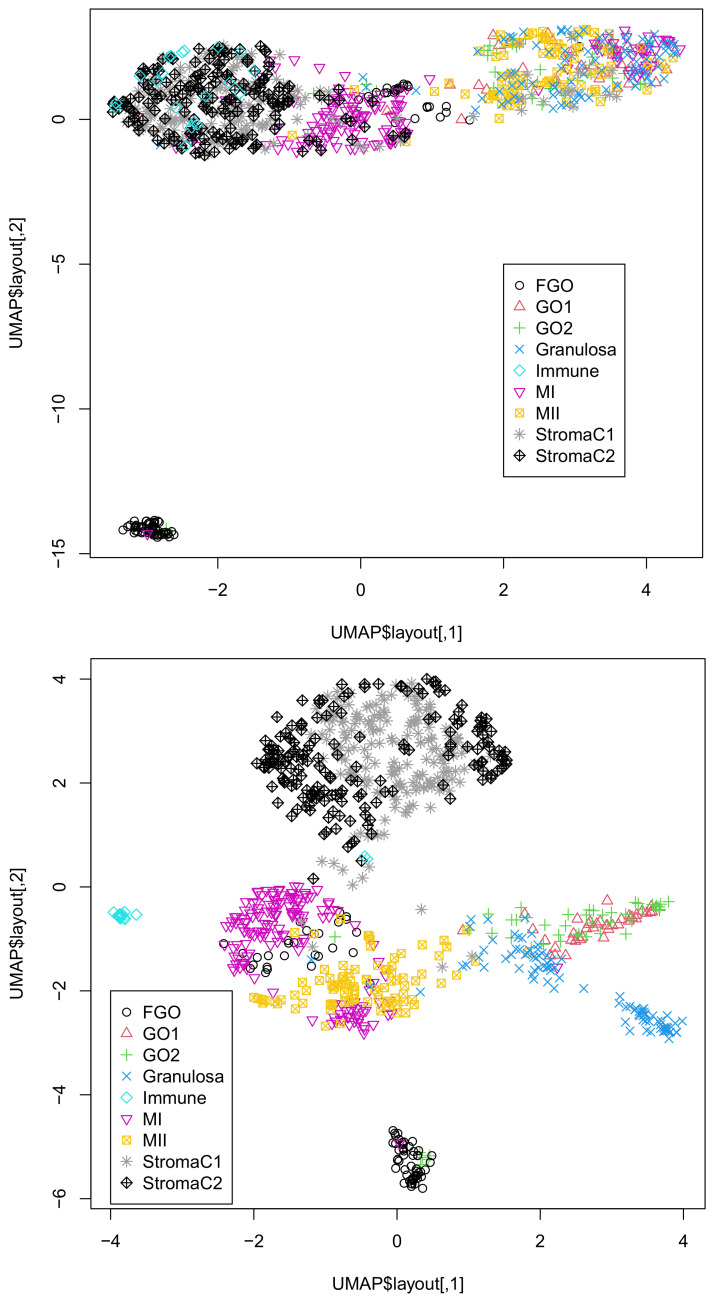
Two-dimensional embedding of singular-value vectors, $u_{\ell_{2}j}$, computed by HOSVD applied to $x_{\ell jk}$ in Dataset 1 (Table 3). Upper: $u_{\ell_{2}j},1\leq \ell_{2}\leq 20$ when only DNA methylation and accessibility (k=2,3) are integrated. Lower: $u_{\ell_{2}j},1\leq \ell_{2}\leq 30$ when all three omics data points (1≤k≤3) are integrated. Default settings other than custom.config$n_neighbors = 100 were used.

To further validate the successful integration of singular-value vectors, we applied UMAP to 20 or 30 singular-value vectors obtained by HOSVD (Figure 1).

It is obvious that the integration of all three omics datasets (lower) was more coincident with classification than that of the integration of the two omics datasets, DNA methylation, and accessibility (upper). This suggests the usefulness of integrating the three omics datasets. In fact, single omics data cannot provide two-dimensional embedding coinciding with classification (Figure S1).

We also attempted to validate biological outcomes when all three omics datasets were integrated. We selected 47 genes associated with adjusted $P_{i}$ less than 0.01, as described in Section 2.7 using $u_{1i}$ because $u_{1i}$ is associated with the largest:

(11) $$\sum_{\ell_{2}}\sum_{\ell_{3}=1}^{3}G^{2}\left(\ell_{1}\ell_{2}\ell_{3}\right),$$

where the summation of $\ell_{2}$ is taken over only 18 $\ell_{2}$s coincident with the classification (Table 3). The selected 47 genes (Data S1) were uploaded to Enrichr [11].

Forty-seven genes were enriched by H3K36me3 based on “ENCODE Histone Modifications 2015”; H3K36m3 is known to play critical roles during oocyte maturation [12]. Forty-seven genes were also targeted by MYC based on “ENCODE and ChEA Consensus TFs from ChIP-X”; Myc is known to play critical roles in oogenesis [13]. Forty-seven genes were also targeted by TAF7 based on “ENCODE and ChEA Consensus TFs from ChIP-X” and “ENCODE TF ChIP-seq 2015”; TAF7 is known to play critical roles during oocyte growth [14]. Forty-seven genes were also targeted by ATF2 based on “ENCODE and ChEA Consensus TFs from ChIP-X”; the expression of ATF2 is known to be altered during oocyte development [15]. This suggests that our strategy correctly captures regulation-related parts (full data of the enrichment analysis are available as Data S1).

### 3.2. Dataset 2

To confirm that the success in the previous section was not accidental, we applied the same procedure to Dataset 2 as well. We obtained $x_{ij1}\in R^{22084\times 758}$, $x_{ij2}\in R^{20106507\times 852}$, and $x_{ij3}\in R^{13627678\times 852}$. SVD was applied to $x_{ijk}$ with L=10, as in Equation (1). For $x_{ijk},k=2,3$, SVD was performed using the irlba function in the irlba package [10] in R because of the large $N_{k},k=2,3$ of as many as ten million. Then, HOSVD was applied to $x_{\ell jk}$, as in Equation (2). Because $N_{1}=758<N_{2}=N_{2}=852$, when HOSVD was applied to $x_{\ell jk}$ composed of all three omics datasets, only 758 single cells shared with all $x_{ijk}$ were considered. As described in the previous section, we first validated the coincidence between singular-value vectors attributed to single cells (Table 4), that is $v_{\ell j}$ and $u_{\ell_{2}\ell k}$, and the classification in Table 2.

**Figure 2:**
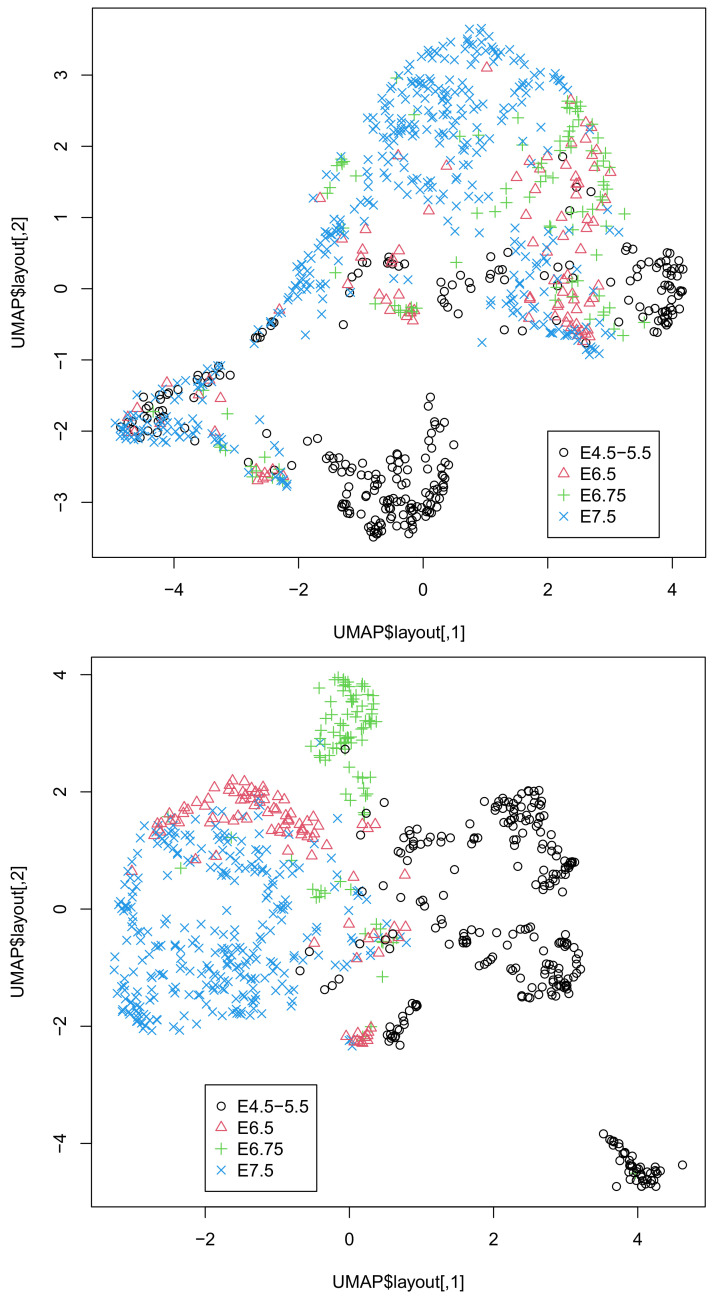
Two-dimensional embedding of singular-value vectors, $u_{\ell_{2}j}$, computed by HOSVD applied to $x_{\ell jk}$ in Dataset 2 (Table 4). Upper: $u_{\ell_{2}j},1\leq \ell_{2}\leq 20$ when only DNA methylation and accessibility (k=2,3) are integrated. Lower: $u_{\ell_{2}j},1\leq \ell_{2}\leq 30$ when all three omics data points (1≤k≤3) are integrated. Default settings other than custom.config$n_neighbors = 100 were used.

The coincidence between the singular-value vectors and the classification in Table 4 was even better than that in Table 3. Thus, it is unlikely that the superior outcome in Table 3 was purely accidental. To further validate the successful integration of singular-value vectors, we applied UMAP to 20 or 30 singular-value vectors obtained by HOSVD (Figure 2).

It is obvious that the integration of all three omics datasets (lower) was more coincident with classification than that of the integration of the two omics datasets, DNA methylation, and DNA accessibility (upper), as can be seen in Figure 2. This again confirms the usefulness of integrating the three omics datasets. In fact, single omics data cannot provide two-dimensional embedding coinciding with classification (Figure S2).

We also attempted to validate biological outcomes when all three omics datasets were integrated. We selected 175 genes associated with adjusted $P_{i}$ less than 0.01, as described in Section 2.7 using $u_{1i}$ because $u_{1i}$ is associated with the largest:

(12) $$\sum_{\ell_{2}}\sum_{\ell_{3}=1}^{3}G^{2}\left(\ell_{1}\ell_{2}\ell_{3}\right)$$

where the summation of $\ell_{2}$ is taken over only 18 $\ell_{2}$s coincident with the classification (Table 4). The selected 175 genes (Data S2) were converted to gene symbols by the DAVID [16] gene ID converter and were uploaded to Enrichr.

One-hundred and seventy-five genes were enriched by H3K36me3 based on “ENCODE Histone Modifications 2015”; H3K36m3 is known to play critical roles during gastrulation [17]. One-hundred and seventy-five genes were also targeted by MYC based on “ENCODE and ChEA Consensus TFs from ChIP-X”; Myc is also known to play critical roles in gastrulation [18]. One hundred and seventy-five genes were also targeted by TAF7 based on “ENCODE and ChEA Consensus TFs from ChIP-X” and “ENCODE TF ChIP-seq 2015”; TAF7 is known to play critical roles during gastrulation [19]. One-hundred and seventy-five genes were also targeted by ATF2 based on “ENCODE and ChEA Consensus TFs from ChIP-X”; the expression of ATF2 is known to be maintained during gastrulation [20]. This suggests that our strategy correctly captured regulation-related parts (full data of the enrichment analysis are available as Data S2).

These two examples, the application to Datasets 1 and 2, demonstrate the usefulness of the present strategy to integrate single-cell multiomics datasets composed of gene expression, DNA methylation, and accessibility.

## 4. Discussion

In this study, we demonstrated the usefulness of our strategy when it was applied to the integrated analysis of single-cell multiomics datasets composed of gene expression, DNA methylation, and DNA accessibility. One might wonder if other more popular methods can achieve similar performance because our strategy is useless if others can perform comparably. There are several advantages of our method, which other methods do not have.

First, we do not have to fill in the missing values with nonzero values. Single-cell measurements are usually associated with a large number of missing values (Table 5).

Although gene expression profiles were associated with a relatively small number of missing components, more than 70 % were missing. For DNA methylation and accessibility, the situation was very difficult to treat. Only a few percentages of components had values, while the rest were missing values. To address this problem, especially for DNA methylation and accessibility, heavy preprocessing is usually required. For example, for Dataset 1, statistical tests were applied and regions associated with significant *p*-values were selected [4], which reduced the number of features attributed to DNA methylation and accessibility. Because such a statistical test automatically filters out regions filled in with missing values, the ratio of nonzero components was also reduced as a result. For Dataset 2, the authors restricted the features to only the most variable ones (typically ∼$10^{3}$) and occasionally filled in missing components with Bayesian models [7]. These procedures inevitably introduce arbitrariness to the outcomes, as preprocessing the data might affect the outcome. In contrast to these arbitrary procedures, our method is almost unsupervised. We did not select any features or fill in the missing values. Despite these fully unsupervised strategies, our results were highly coincident with the classification (Table 3 and Table 4 and Figure 1 and Figure 2). From this perspective, our strategy is superior to the other methods.

Second, our method can deal with massive datasets. For example, although integrated analysis of multiomics data was performed using multiomics factor analysis (MOFA) [21] in the original studies [4,7] of Datasets 1 and 2, MOFA cannot accept $x_{ijk}$ in this study as inputs because MOFA does not implement sparse matrix architecture. During the computation of MOFA, zero values must be filled in with nonzero values to evaluate the convergence; this results in a dense matrix that cannot be stored in the computer memory because the number of components of DNA methylation and accessibility is too large to store them as they are (Table 5). In our computation, we can apply SVD to these large datasets while keeping them in a sparse matrix format using the irlba package implemented in R. SVD not only reduces the number of features to *L*, but also fills in missing values. Thus, we can manage a large matrix as in our implementation.

Third, our method is free from the dividing weight between multiomics datasets; how to weigh individual omics data must be decided based on some criteria outside the datasets available. Nevertheless, in our implementation, the weight of individual omics data is represented by $u_{\ell_{3}k}$, which is automatically decided by simply applying HOSVD to a multiomics dataset. Thus, from this perspective, our strategy is outstanding.

Although we showed that the integration of all three omics data was superior to that of the integration of DNA methylation and accessibility (Figure 1 and Figure 2), one might wonder if the integration of gene expression and DNA methylation or DNA accessibility might be comparable to that of all thee omics datasets. In order to deny this possibility, we also considered these combinations of two of the three omics datasets (Figures S3 and S4). Although the integration of gene expression and DNA accessibility in Dataset 1 (Figure S3B) is comparable to that of all three omics data, neither integration of gene expression and DNA methylation (Figure S4A) nor that of gene expression and DNA accessibility (Figure S4B) is comparable to that of all three omics data in Dataset 2. Thus, it is obvious that only the integration of the three omics datasets can give us UMAP embedding coincident with the classification regardless of the dataset considered.

As for the comparisons with other methods, as mentioned above, no methods implemented with a sparse matrix architecture and applicable to multiomics datasets exist to our knowledge. Thus, we could not compare our performance to other methods.

Prospective uses of our methods are as follows. First of all, it can integrate gene expression profiles, DNA methylation, and accessibility in single-cell measurements without applying preprocessing; this enables researchers to obtain reasonable results without struggling to convert raw data into treatable formats. In addition to this, since it can save the memories required for analyzing single-cell multiomics datasets, more researchers who do not have massive computational facilities can analyze massive single-cell measurements.

## 5. Conclusions

In this study, we proposed a method for applying TD-based unsupervised FE to single-cell multiomics datasets composed of gene expression, DNA methylation, and DNA accessibility. Together with UMAP, the proposed method successfully integrated a multiomics dataset and generated a two-dimensional embedding of single cells coincident with the classification. The implementation requires neither filling missing values nor massive CPU memory to store multiomics datasets of single cells and can deal with DNA methylation and accessibility with ten million features. The present implementation is very promising and can be a de facto standard method to integrate single-cell multiomics datasets composed of gene expression, DNA methylation, and DNA accessibility.

## Figures and Tables

**Table 1:** The number of single cells within individual cell types included in Dataset 1.

FGO	GO1	GO2	Granulosa	Immune	MI	MII	StromaC1	StromaC2
81	40	46	93	20	155	90	189	185

**Table 2:** The number of single cells at four embryonic time points included in Dataset 2. For E7.5, the gene expression profiles of 296 single cells were measured.

E4.5-5.5	E6.5	E6.75	E7.5
267	98	97	390 (296)

**Table 3:** Number of singular-value vectors coincident with classification shown in Table 1.

	SVD ($v_{\ell \mathrm{jk}}$)			HOSVD ($u_{\ell_{2}j}$)	
Adjusted	Gene	DNA	DNA	DNA Methylation	
*p*-Value	Expression	Methylation	Accessibility	and Accessibility	All
<0.01	10	7	1	10	18
≥0.01	0	3	9	10	12

**Table 4:** Number of singular-value vectors coincident with the classification shown in Table 2.

	SVD ($v_{\ell \mathrm{jk}}$)			HOSVD ($u_{\ell_{2}j}$)	
Adjusted	Gene	DNA	DNA	DNA Methylation	
*p*-Value	Expression	Methylation	Accessibility	and Accessibility	All
<0.01	10	7	5	10	18
≥0.01	0	3	5	10	12

**Table 5:** Number of single cells, features, nonzero components, and their ratios.

Numbers	Expression	DNA Methylation	DNA Accessibility
	Dataset 1		
single cells	899	899	899
features	26,500	26,438,807	15,478,375
total components	$2.38\times 10^{7}$	$2.38\times 10^{10}$	$1.39\times 10^{10}$
nonzero components	$6.76\times 10^{6}$	$5.50\times 10^{8}$	$3.85\times 10^{8}$
the ratio of nonzero components	0.28	0.02	0.03
	Data set 2		
single cells	758	852	852
features	22,084	20,106,507	13,627,678
total components	$1.67\times 10^{7}$	$1.71\times 10^{10}$	$1.16\times 10^{10}$
nonzero components	$4.87\times 10^{6}$	$6.96\times 10^{8}$	$7.87\times 10^{8}$
the ratio of nonzero components	0.29	0.04	0.07

## Data Availability

The data used in this study are available in GEO ID GSE154762 and GSE121708. A sample of the R source can be found at https://github.com/tagtag/scMultiR (accessed on 14 September 2021).

## Supplementary Materials

The following are available online at https://www.mdpi.com/article/10.3390/genes12091442/s1, Figure S1: UMAP embedding of single omics data for data set 1, Figure S2: UMAP embedding of single omics data for data set 2, Figure S3: UMAP embedding of integration of two omics data for data set 1, Figure S4: UMAP embedding of integration of two omics data for data set 2.

## Abbreviations

The following abbreviations are used in this manuscript:

BH	Benjamini–Hochberg
FE	feature extraction
HOSVD	higher-order singular-value decomposition
HTS	high-throughput sequencing
MOFA	Multi-Omics Factor208Analysis
SVD	singular-value decomposition
TD	tensor decomposition
