# Supplementary material for: Tensor-Decomposition-Based Unsupervised Feature Extraction in Single-Cell Multiomics Data Analysis
Source: Genes (Basel). 2021 Sep 18;12(9):1442. doi: 10.3390/genes12091442 (PMC8468466; doi:10.3390/genes12091442)
Supplement: Supplementary file 1 [file genes-12-01442-s001.zip › Supplementary_Figures.pdf]

(A)

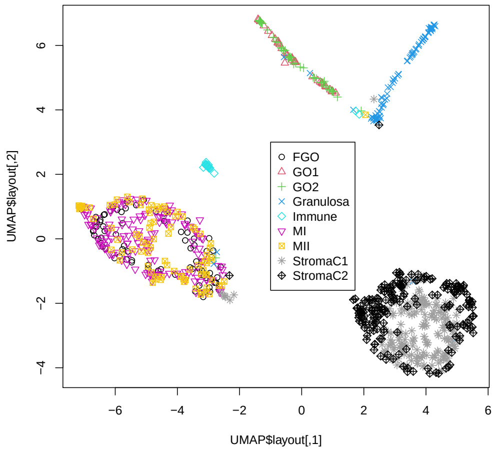

(B)

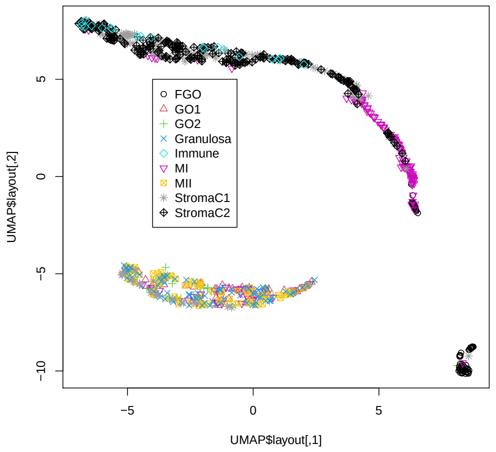

(C)

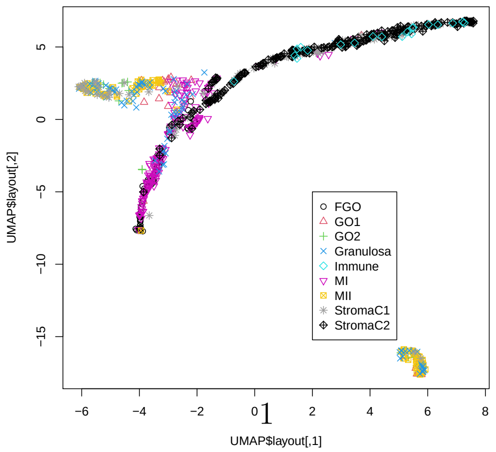

Figure S1: UMAP embedding of single omics data for data set 1. (A) gene expression (B) DNA methylation (C) DNA accessibility. Default setting other than custom.config$n_neighbors=100 are used.

(A)

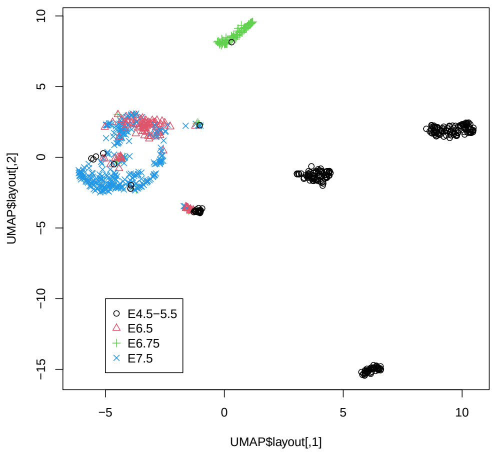

(B)

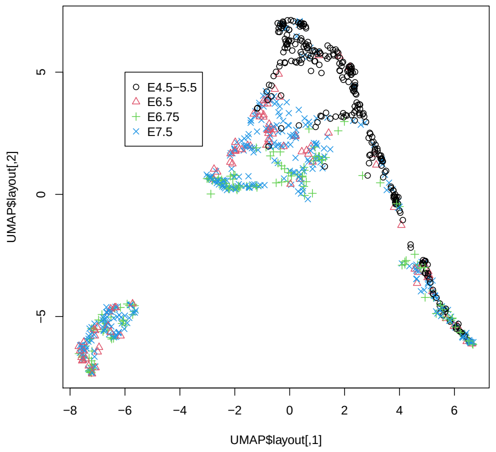

(C)

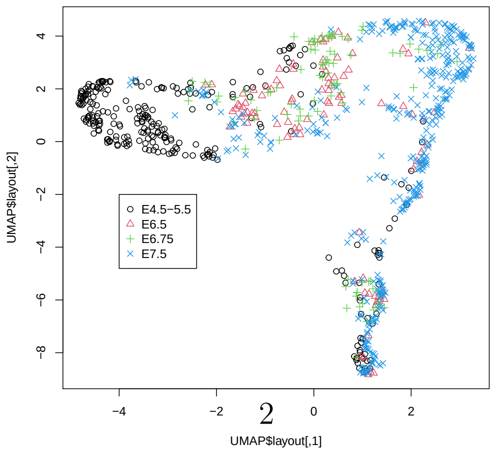

Figure S2: UMAP embedding of single omics data for data set 2. (A) gene expression (B) DNA methylation (C) DNA accessibility. Default setting other than custom.config$n_neighbors=100 are used.

(A)

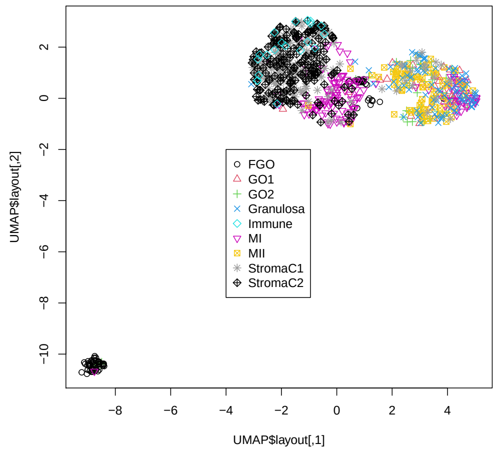

(B)

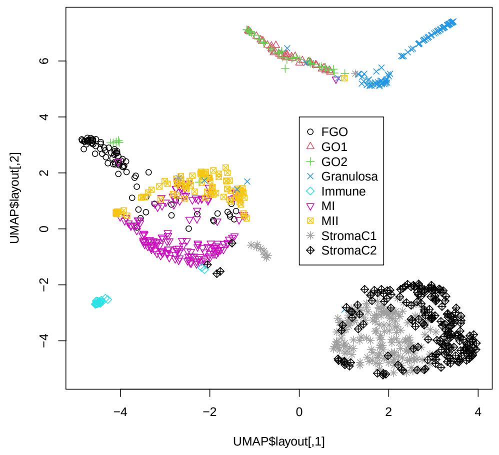

Figure S3: Two-dimensional embedding of singular value vectors, $u_{\ell_2 j}$, computed by HOSVD applied to $x_{\ell jk}$ in dataset 1. (A) $u_{\ell_2 j}, 1 \leq \ell_2 \leq 20$ when only gene expression and DNA methylation ($k = 1, 2$) are integrated. (B) $u_{\ell_2 j}, 1 \leq \ell_2 \leq 20$ when only gene expression and DNA accessibility ($k = 1, 3$) are integrated. Default settings other than custom.config$n_neighbors=100 were used.

(A)

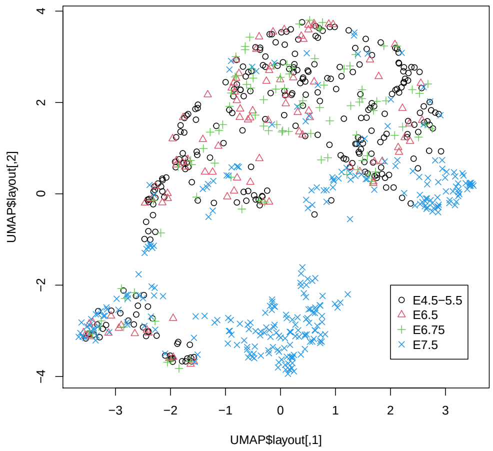

(B)

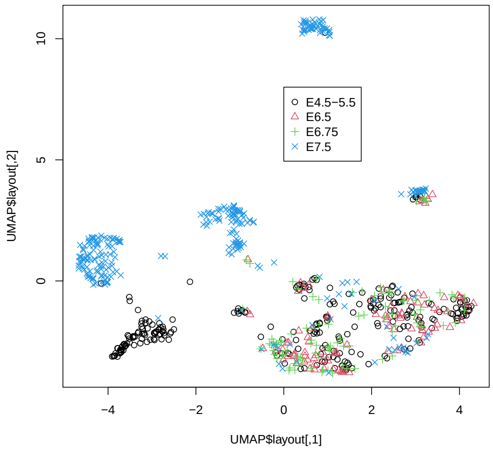

Figure S4: Two-dimensional embedding of singular value vectors, $u_{\ell_2 j}$, computed by HOSVD applied to $x_{\ell jk}$ in dataset 2. (A) $u_{\ell_2 j}, 1 \leq \ell_2 \leq 20$ when only gene expression and DNA methylation ($k = 1, 2$) are integrated. (B) $u_{\ell_2 j}, 1 \leq \ell_2 \leq 20$ when only gene expression and DNA accessibility ($k = 1, 3$) are integrated. Default settings other than custom.config$n_neighbors=100 were used.
